# Protective antigenic sites in respiratory syncytial virus G attachment protein outside the central conserved and cysteine noose domains

**DOI:** 10.1371/journal.ppat.1007262

**Published:** 2018-08-24

**Authors:** Jeehyun Lee, Laura Klenow, Elizabeth M. Coyle, Hana Golding, Surender Khurana

**Affiliations:** Division of Viral Products, Center for Biologics Evaluation and Research (CBER), FDA, Silver Spring, MD, United States of America; University of Georgia, UNITED STATES

## Abstract

Respiratory syncytial virus (RSV) is the major cause of lower respiratory tract disease in infants. Previously, we elucidated the antibody repertoire following primary RSV infection in infants. Whole genome-fragment phage display libraries (GFPDL) expressing linear and conformational epitopes from RSV bound 100-fold more phages within attachment protein (G) following primary RSV infection. The G-reactive epitopes spanned the N- and C-termini of G ectodomain, in addition to the central conserved domain (CCD). In the current study, we examined the contribution of antigenic regions of G outside of the CCD to RSV-specific immunity. We evaluated the immunogenicity, neutralization and protective efficacy of all RSV-G antigenic sites identified following primary RSV infection using recombinant *E*. *coli* expressed G ectodomain (REG), CCD-deleted G ectodomain (REG ΔCCD), N- and C-terminal G subdomains, and antigenic site peptides. The REG ΔCCD, N- and C-terminal subdomains and peptides generated antibody titers in rabbits and mice that bound fully glycosylated Recombinant Mammalian expressed G ectodomain (RMG) and intact RSV virion particles but minimal *in vitro* neutralization titers compared with the intact G ectodomain. Vaccinated mice were challenged intranasally with RSV-A2 Line 19F. Viral replication in nasal cavity and lungs was significantly reduced in vaccinated animals compared to unimmunized controls. Control of viral loads post-RSV challenge correlated with serum antibody binding to the virus particles. In addition, very low Th2/Th1 cytokine ratios were found in the lungs of REG ΔCCD vaccinated mice after challenge. These data demonstrate the presence of multiple protective sites in RSV G protein outside of the CCD that could contribute to the development of a bacterially produced unglycosylated G protein as safe and protective vaccine against RSV disease.

## Author summary

A vaccine against RSV that provides protection without potential for disease enhancement is required. The G attachment protein represents an important candidate for inclusion in an effective RSV vaccine. However, the contribution of different antigenic sites to protection against RSV is not completely understood. We evaluated the protective efficacy of recombinant unglycosylated RSV-G protein vaccine produced in *E*. *coli* (REG) vs. CCD-deletion (REG ΔCCD). We also investigated immunogenicity and protective efficacy of all antigenic sites identified in post-primary infection infant sera using GFPDL that includes N- and C-terminal G subdomains, and linear peptides. The REG ΔCCD, N- and C-terminal subdomains and peptides generated antibody titers in rabbits and mice. Vaccinated mice challenged intranasally with RSV demonstrated significant reduction of viral replication in the nasal cavity and lungs. Our study highlights the safety and immunogenicity of recombinant G protein as economical protective vaccine against RSV disease.

## Introduction

Respiratory syncytial virus (RSV) is the major cause of lower respiratory tract disease among infants and children globally [1] [2] [3]. Hospitalizations for respiratory tract disease among young children, especially in less than one year old, is most often attributed to RSV infection[4] [5]. Furthermore, despite the development of immunity following RSV infection during childhood, individuals remain susceptible to RSV upper respiratory tract reinfection life-long[6, 7] [8].

RSV isolates can be classified into two antigenically distinct groups (A and B) with genetic differences occurring mostly in the attachment glycoprotein G (47% heterogeneity at the amino acid level) and to a lesser degree in the fusion protein F (9%) [9]. In addition, continuous evolution of RSV generates diversity primarily in the G gene[10] [11]. Heterologous RSV strains are the main cause of re-infections, and homologous RSV strains are observed less frequently [12] [13]. However, while there are instances of evolution, e.g. the RSVs with duplications in the G gene, there are also cases of same genotype reappearing over many years. Even though F specific antibodies have been reported to contribute to majority of virus neutralization measure *in vitro* PRNT assays, the relative contribution of F and G specific antibodies to protection *in vivo* is not completely understood. A recent study by Capella *et al* found that higher concentrations of pre-F and G antibodies (but not post-F antibodies) were associated with lower clinical disease severity in infants and young children (< 2yr) [14].

In a previous study, we generated gene fragment phage display libraries (GFPDL) for the RSV surface proteins F and G to elucidate the complete antibody epitope repertoire in serum samples from infants either prior to (<9 months) or after primary and early RSV infection (15–18 months) [1]. Primary RSV infection predominantly resulted in an increase of G specific binding antibodies as observed by 100-fold increase in the number of phages that bound to the post-RSV infection sera compared with pre-infection sera. Bound phages displayed epitopes that spanned most of the ectodomain of RSV-G with two large conformationally dependent antigenic regions flanking the CCD motif in addition to the CCD [1].

In the current study, we evaluated the immunogenicity of the G-ectodomain lacking part of the central conserved domain (CCD) and the cysteine noose, as well as the individual G-subdomains (N- and C- termini), and G-derived peptides previously identified using GFPDL analysis of post-RSV infection infant sera by immunization of rabbits and mice using virus plaque reduction neutralization test (PRNT), several binding assays including recombinant mammalian cell produced G ectodomain RMG), RSV A2 virions, and recombinant CX3CR1 competition assay. The protective efficacy of these antigenic sites was determined in mouse challenge studies with RSV-A2 line 19F expressing firefly luciferase [rRSV-A2-L19-FFL]. Viral loads in the nasal cavity and lungs were inferred using fluorescence measurements obtained via whole body live imaging as previously described [15] in addition to plaque forming units (PFU) in the lungs. We found that animals vaccinated with REG ΔCCD, G-subdomains, and G-peptides had significantly lower viral loads after RSV challenge than unimmunized controls. Lung viral loads inversely correlated with RSV A2 virion binding antibody titers (but not with *in vitro* neutralization titers). Low lung pathology and low Th2/Th1 cytokine ratios was observed in all vaccinated-challenged groups. Therefore, several antigenic sites apart from the CCD motif in the G protein provide protective immunity against RSV.

## Results

### Generation of CCD deleted RSV G protein and G-subdomains in *E*. *coli*

**Fig 1:**
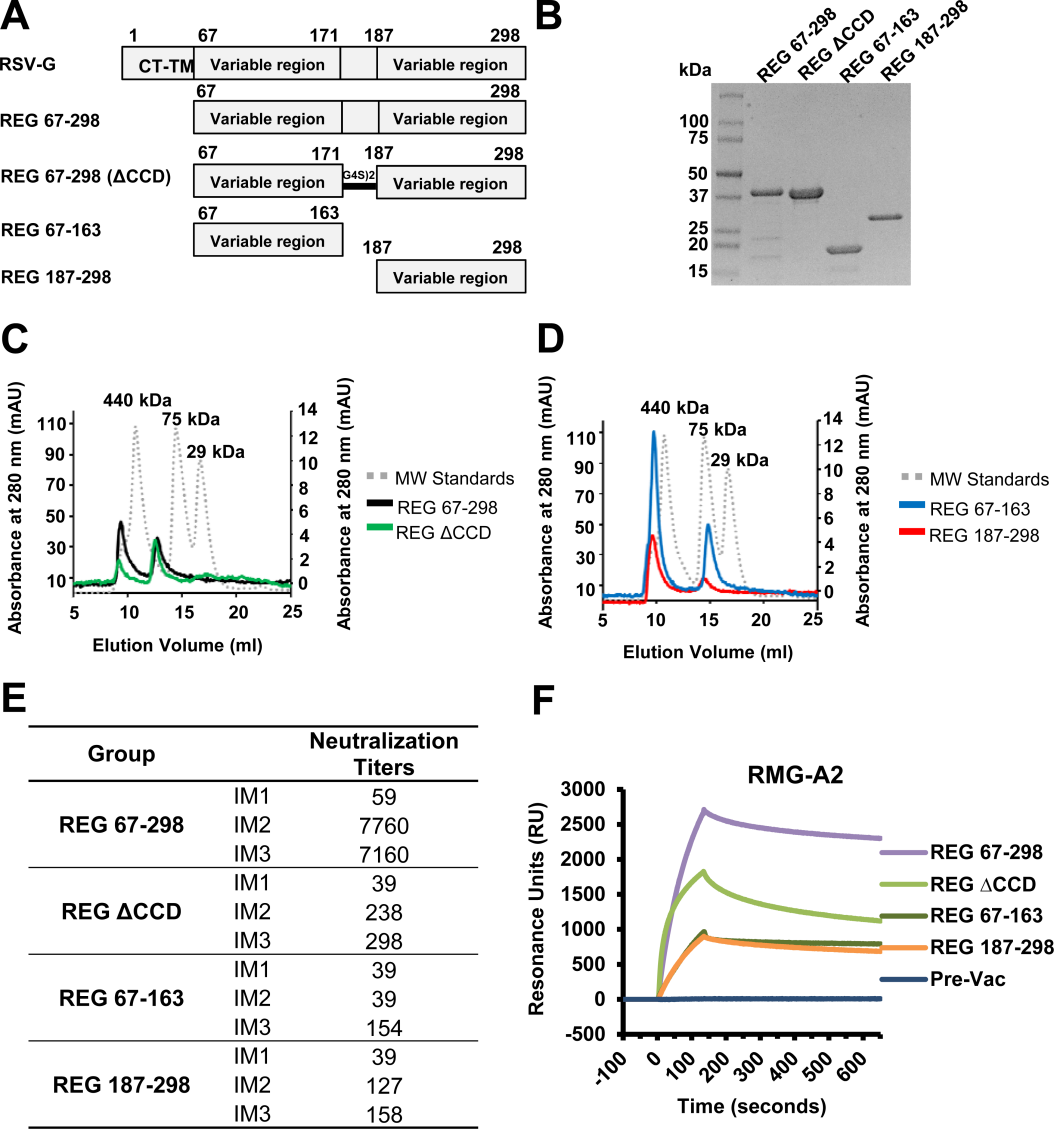
Recombinant G proteins produced in *E*. *coli*. (A-B) Schematic representation of the RSV G protein and subdomains. RSV G ectodomain lacks the cytoplasmic and transmembrane domains (CT-TM) and is not glycosylated (REG) since it is produced in *E*. *coli*. REG 67–298, REG 67–298 with aa 172–186 deleted (REG ΔCCD), REG N-terminus (aa 67–163), and REG C-terminus (aa 187–298), all containing 6x His tag at C-terminus, were expressed in *E*. *coli* and purified using a Ni-NTA column, and subjected to SDS-PAGE under reducing conditions. (C-D) Superdex 200 10/300 gel filtration chromatography of RSV G proteins produced in the bacterial system (REGs). Elution profiles of purified REG 67–298 (indicated by black line) and REG ΔCCD (green) (C), REG 67–163 (blue) and REG 187–298 (red) (D) are overlaid with molecular weight standards (gray dotted line). (E) Neutralizing antibody response following REG 67–298, REG ΔCCD, REG 67–163, and REG 187–298 immunizations in rabbits by PRNT assay. Serum samples were collected from individual rabbits 8 days after the first (IM1), second (IM2), and third (IM3) immunizations, and were tested for neutralization by PRNT against the RSV A2 strain. Neutralizing antibody titers represent 50% plaque inhibition. (F) SPR analysis of post-third vaccination serum antibodies (diluted 10-fold) binding to fully glycosylated G ectodomain RMG from RSV-A2 [16].

Our earlier findings with post primary infection in plasma from infants, demonstrated very broad epitope repertoire spanning the entire G ectodomain. In the current study, we investigated the contributions of antigenic regions outside of the conserved central domain (CCD; aa residues 172–186) of G to RSV-specific immunity. As a comparator, we used a G-ectodomain protein (residues 67–298) of RSV-A2 containing the CCD motif produced using *E*. *coli* (REG 67–298), which was previously found to generate protective immunity in mice and cotton rats [16, 17]. We evaluated the immunogenicity of a CCD-deleted G-ectodomain [REG ΔCCD; with residues 172–186 replaced by a (Gly_4_Ser)_2_ linker], and two large G subdomains covering the N-terminus (REG 67–163) and C-terminus (REG 187–298) upstream and downstream of the CCD, respectively, that were identified as immunodominant in the epitope profiling of post-RSV primary infection human sera (Fig 1 panels A-B). Size exclusion chromatography (SEC) profiles of the four REG proteins using Superdex 200 gel filtration are illustrated in Fig 1 panels C-D. All recombinant G proteins ran as two distinct peaks likely representing tetramers and higher molecular weight oligomers.

### CCD is required for *in vitro* neutralization antibody response following rabbit immunization

Rabbits were immunized three times intramuscularly (i.m.) with 100 μg of purified REG protein combined with Emulsigen adjuvant, bled 8 days after each immunization, and sera evaluated in plaque reduction neutralization assay (PRNT) in the presence of guinea pig complement (GPC). GPC was added to increase the sensitivity of the neutralization assay, as previously we have shown that GPC specifically promotes RSV-A2 virus neutralization by anti-G but not anti-F specific antibodies *in vitro* [18]. As expected, the intact ectodomain (REG 67–298) was highly immunogenic, with peak neutralization titers measured after the second vaccination (Fig 1E). The REG ΔCCD and the two large fragments elicited modest or low neutralization titers after the second or third vaccinations, respectively (Fig 1E).

Since *in vitro* PRNT assays may not detect all antibodies, we also evaluated the binding of immune sera (post-third vaccination) to fully glycosylated G protein from RSV-A2 strain produced in mammalian 293 cells (RMG-A2) [16]. All four recombinant proteins generated antibodies in rabbits that bound to fully glycosylated G protein (Fig 1F). The REG 67–298 immune sera demonstrated the highest peak binding (2,500 RU), followed by REG ΔCCD immune sera (1,600 RU), and by the immune sera generated against the N-terminal and C-terminal domains. The hierarchy of binding to the glycosylated G recapitulated the PRNT virus neutralization titers for the same sera. These findings suggested that while the CCD region is key for generation of measurable neutralizing antibodies in the PRNT assay, there are immunological targets outside the CCD that elicit antibodies that can bind to the fully glycosylated G attachment protein.

### Murine challenge studies demonstrate protective efficacy of antigenic regions apart from CCD

**Fig 2:**
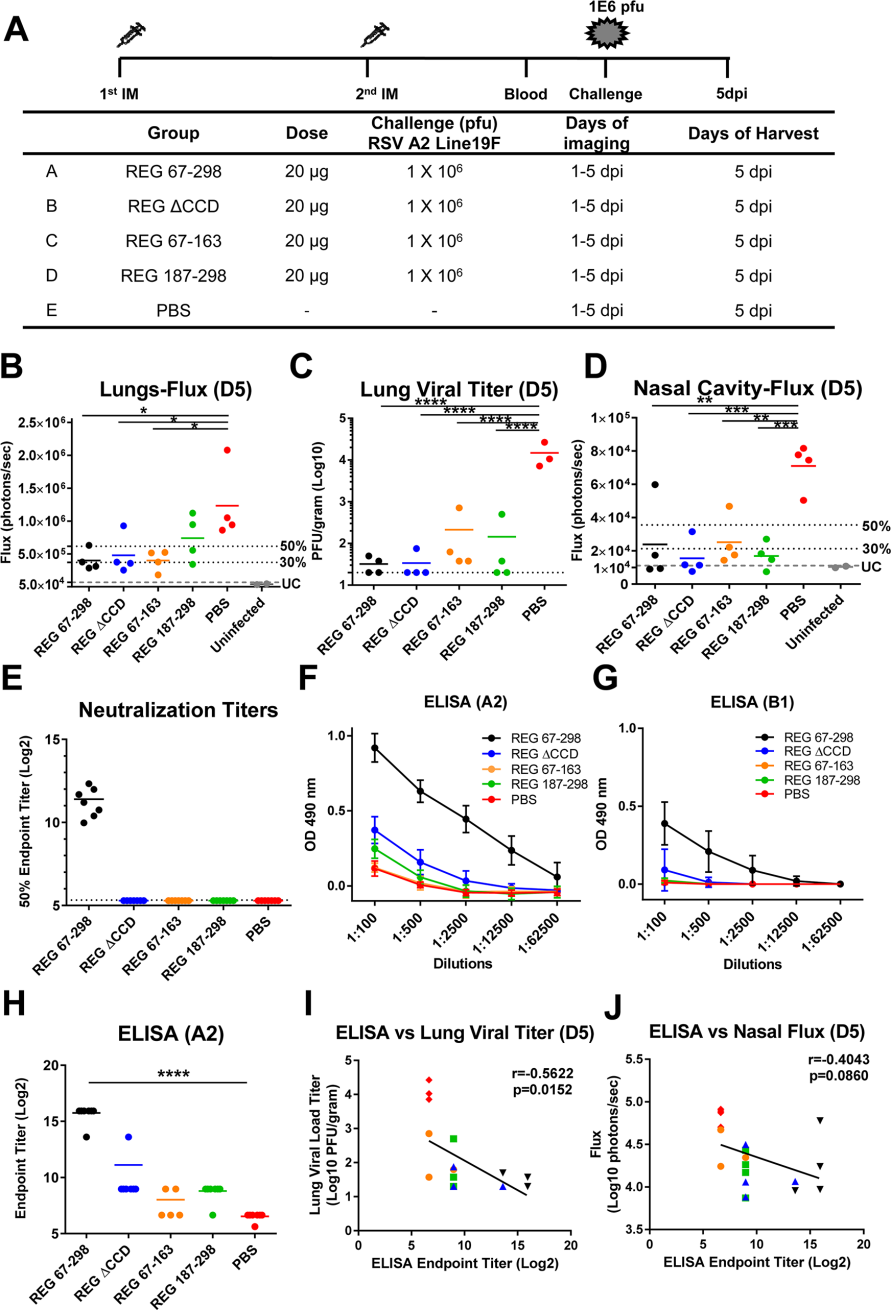
Recombinant G protein immunization and challenge study in mice. (A) Schematic representation of mouse immunization and challenge schedule. BALB/c mice (N = 8 per group) were immunized i.m. with 20 μg of REG 67–298, REG ΔCCD, REG 67–163, and REG 187–298 from RSV strain A2 with Emulsigen adjuvant, or with PBS as a control. After the second immunization, blood was collected from the tail veins. Then, mice were challenged intranasally with 10^6^ PFU of RSV rA2-Line-19F-FFL expressing the homologous G protein. *In vivo* imaging of lungs and the nasal cavity was performed daily for 5 days following RSV infection. Mice were sacrificed on day 5 post-challenge, when lungs and blood were collected. (B and D) Live whole body imaging was performed to detect firefly luciferase activity in the lungs (B) and nasal cavity (D). Graphs represent the quantification of total flux (photons/sec) on day 5 post challenge as previously described [15]. 50% and 30% of the mean flux value of the PBS control group is indicated by the two dotted lines. The lowest grey lines (UC) represents the average flux values of the uninfected control mice (5.23 x 10^4^ for lungs and 1.11 x 10^4^ for nasal cavity). (C) Lung viral loads at 5 days post- viral challenge as measured by plaque assay. (E) Serum samples were collected from individual mice after the second immunization and were tested for neutralization by PRNT against RSV A2 strain in A549 cells. Neutralizing antibody titers represent 50% inhibition of plaque numbers. The average for each group is indicated by a horizontal line. The dotted lines indicate cutoff values based on the 1:20 dilution of sera used in PRNT. (F-G) The same serum samples were analyzed by ELISA using plates coated with purified RSV rA2-Line19F-FFL (F) or RSV-B1 (G) virion particles. The serum samples were serially diluted and the detection of antibodies were measured by absorbance at 490 nm. The mean absorbance + SD for each dilution is indicated. (H) End-point titers of the serum samples were determined as the reciprocal of the highest dilution providing an optical density (OD) twice that of the negative control. (I-J) Correlation between endpoint titer (Log2) and viral load measured by plaque assay in lungs on day 5 post-challenge (I) or bioluminescence flux signal in the nasal cavity on day 5 post-challenge (J). REG 67–298 group is indicated by black triangles, REG ΔCCD by blue triangles, REG 67–163 by yellow circles, REG 187–298 by green squares, and PBS by red rhombuses. Inverse Spearman correlations were observed between endpoint titers measured by ELISA vs. Lung viral loads (r = -0.5622; *p = 0*.*0152*) (I) and vs. Nasal Cavity Fluxes (r = -0.4043; *p = 0*.*0860*) (J). Statistical significance was tested by one-way ANOVA and Bonferroni multiple-comparison tests. ****, *p* <0.0001; ***, *p* <0.001; **, *p* <0.01 *, *p* <0.05.

Mice were immunized intramuscularly (i.m.) at day 0 and day 20 with 20 μg of purified REG proteins combined with Emulsigen adjuvant. Blood was collected from the tail vein on days 0, 14, and 30. On day 34, mice were inoculated intranasally (i.n.) with 10^6^ PFU of RSV rA2-Line19F-FFL containing homologous RSV-A2 G protein sequence identical to the immunizing REG protein [15] (Fig 2A). Previously, we described the applicability of live imaging for following RSV replication and dissemination from the nasal cavity to the lungs, and demonstrated a strong correlation between bioluminescence flux units and viral loads measurements by PFU in the lungs of infected animals [15]. All animals vaccinated with REG 67–298 (intact G-ectodomain) completely controlled virus replication in the lung as measured by either live imaging (Fig 2B) or plaque assay (Fig 2C) compared to sham (PBS) vaccinated animals. Surprisingly, most the animals immunized twice with REG ΔCCD were also capable of controlling virus replication in the lungs by day 5 post-challenge (Fig 2B and 2C). Unexpectedly, some of the animals immunized with the N-terminus REG 67–163, or the C-terminus REG 187–298 also showed significant protection against viral replication in lungs as determined by either Flux or PFU measurements (Fig 2B and 2C). In addition to lungs, the live imaging allowed us to measure viral loads in the nasal cavities. All groups of vaccinated animals showed significant reduction in nasal viral loads compared with the PBS control group (Fig 2D).

In search for correlates of protection, we evaluated the immune sera (pre-challenge) in virus neutralization and virus particle–binding ELISA. Only sera from mice vaccinated with the entire G-ectodomain (REG 67–298) had detectable virus neutralization activity *in vitro* (Fig 2E). As such, the mechanism of protection was not apparent for the REG proteins without CCD and for the N- and C- domains. Several anti-G specific MAbs including 131-2G have been defined that do not neutralize in classical A549 based virus neutralization assay however provide significant protection in animal challenge studies [19, 20]. Therefore, we evaluated total antibody binding to RSV particles by ELISA. We have confirmed in earlier studies that the virus ELISA express relevant protective epitopes (including conformational epitopes) and can capture monoclonal and polyclonal antibodies with protective titers. However, it is possible that coating of virus particles (virions) on microtiter plates in ELISA may not accurately represent some aspects of structurally intact virions. Antibody binding to RSV A2 virions was highest in immune sera from animals vaccinated with the entire RSV-G ectodomain (REG 67–298) (Fig 2F). However, some binding to virus particles was also observed with sera from REG ΔCCD, and to a lesser degree with sera from animals vaccinated with the C-terminus domain (REG 187–298) and even weaker binding with sera from the N-terminal domain immunized mice (REG 67–163). The binding antibody endpoint titers to RSV A2 virions are shown in Fig 2H. We also measured binding to RSV B1 virion particles. As expected, sera from REG 67–298 vaccinated animals bound RSV B1 virions in ELISA although to a lower level compared to RSV-A2 virions, in agreement with our earlier study [16] (Fig 2G). In contrast, the sera from the other three groups did not show significant binding to RSV B1 virions.

On day 5 post-challenge, the viral loads (as measured by plaque assay) in the lungs (Fig 2C) and nasal cavity (measured by live imaging) (Fig 2D) inversely correlated with virion binding antibody endpoint titers (Fig 2I and 2J) (r = -0.5622 and r = -0.4043, respectively) that reached statistical significance for the lungs (*p < 0*.*0152*). The results show that *in vivo* challenge studies are more sensitive than *in vitro* assays for detection of protective activity of RSV G targeting immune mechanisms.

**Fig 3:**
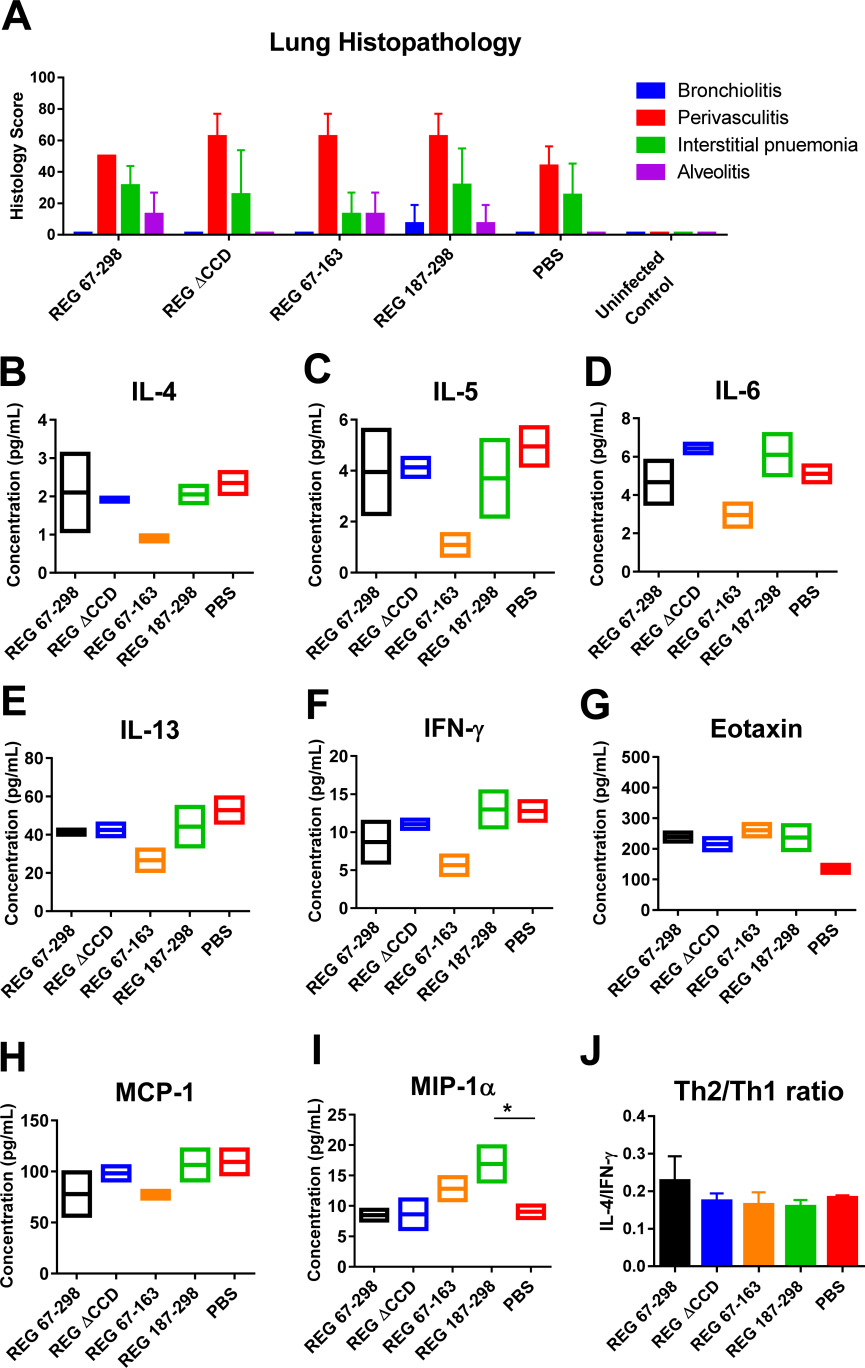
Histopathology and cytokine/chemokine levels in the lungs of animals vaccinated with the REG 67–298, REG ΔCCD, REG 67–163, and REG 187–298 at day 5 following RSV challenge. (A) Lung tissues were collected at 5 days post-challenge and were stained with hematoxylin and eosin. Individual lungs were scored for pulmonary inflammation: bronchiolitis (mucous metaplasia of bronchioles), perivasculitis (inflammatory cell infiltration around the small blood vessels), interstitial pneumonia (inflammatory cell infiltration and thickening of alveolar walls), and alveolitis (cells within the alveolar spaces). Slides were scored blindly using a 0–4 severity scale. The scores were subsequently converted to a 0–100% histopathology scales. (B-I) Levels of cytokines and chemokines in lung homogenates from day 5 post challenge were measured in a Bio-Plex Pro mouse cytokine assay. Values are cytokine/chemokine concentrations, expressed in pictograms per milliliter. The mean for each group is shown. (J) Ratio of the observed concentrations of IL-4 vs. IFNγ shown in panels B and F. Statistical significance was tested by one-way ANOVA and Bonferroni multiple-comparison tests. *, *p* <0.05.

In addition to viral loads, lung pathology (bronchiolitis, perivasculitis, interstitial pneumonia, and alveolitis) was evaluated for all mice on day 5 post-RSV challenge (Fig 3A). The histology scores for vaccinated animals were not significantly different from the sham vaccinated (PBS) control group. To further explore the possibility of perturbation to the local cytokine milieu, the lung extracts were tested against a cytokines/chemokines multiplex panel (Fig 3B–3I). Levels of most cytokines/chemokines were similar between the vaccinated animals and were not significantly different from the sham vaccinated (PBS) control group following RSV challenge. However, a non-significant elevation of Eotaxin in the vaccinated groups was observed compared with the control group (Fig 3G), and an elevated MIP-1α levels in the group vaccinated with the C-terminus antigenic domain (REG 187–298) (Fig 3I). But there were no significant differences in the levels of Th1 and Th2 cytokines and the Th2/Th1 ratio (Fig3J) [16].

### Immunization of rabbits and mice with short antigenic site peptides derived from the N- and C- termini of G-ectodomain

**Fig 4:**
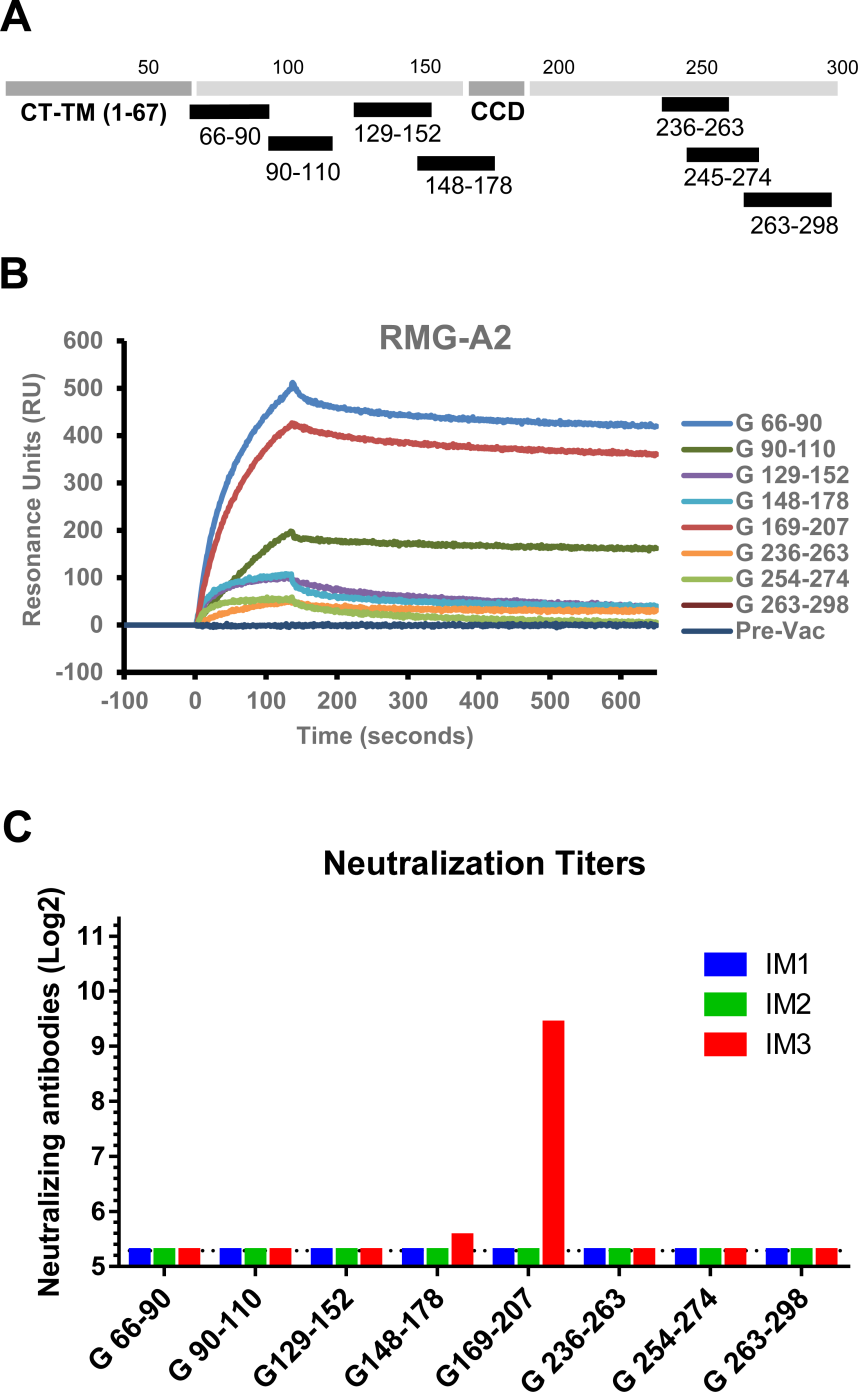
Immunization of rabbits with RSV G peptides. (A) Schematic diagram of RSV G peptides, conjugated to KLH, that were used to immunize rabbits, combined with Emulsigen adjuvant. (B) SPR analysis of post-third vaccination serum antibodies (diluted 10-fold) to RMG from RSV A2 strain. (C) Virus neutralization titers following first, (IM1), second (IM2) and third (IM3) vaccinations. Titer is shown for 50% reduction in number of plaques.

Next we synthesized peptides representing linear antigenic sites that were identified in human post-primary RSV infection in infants [1]. These peptides were conjugated to KLH and mixed with Emulsigen for vaccination of rabbits and mice (Fig 4A).

The rabbit polyclonal sera obtained after two peptide vaccine doses bound to glycosylated RMG protein in SPR. However, antibody binding titers were 3 to 10-fold lower than antibody binding observed following immunization with REG ΔCCD (Fig 4B vs. Fig 1F). Interestingly, the peptide that elicited the highest RMG-binding titer was found in rabbits immunized with the N-terminal G peptide (aa 66–90). However, only peptide G169-207 that overlapped the CCD region elicited neutralizing antibodies after three immunizations (Fig 4C).

**Fig 5:**
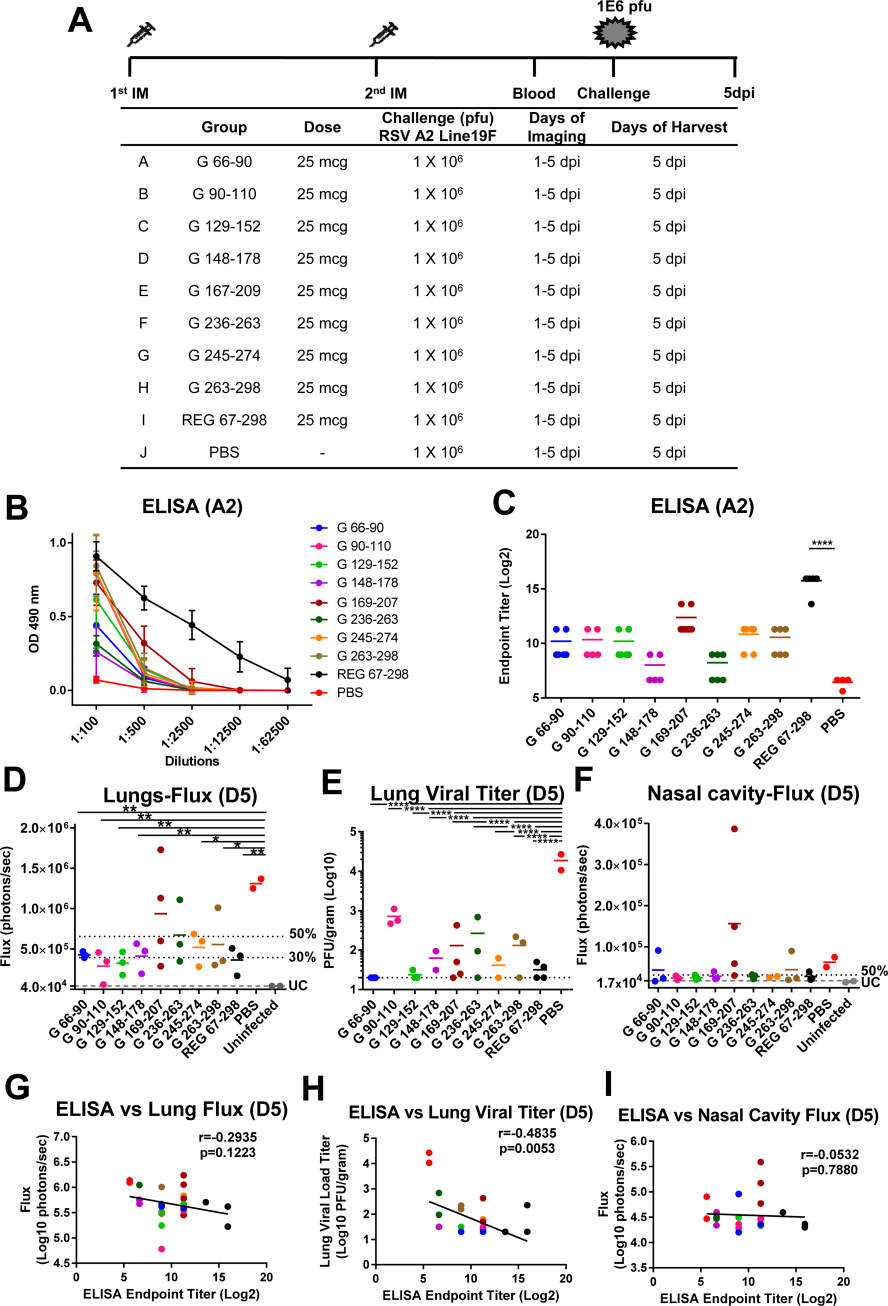
RSV G peptide immunization and challenge study in mice. (A) Schematic representation of mouse immunization and challenge schedule. BALB/c mice (N = 6 per group) were immunized i.m. with 25 μg of KLH-conjugated peptides: G 66–90, G 90–110, G 129–152, G 148–178, G 169–207, G 236–263, G 245–274, G 263–298 or with 20 μg of RSV strain A2 REG 67–298 with Emulsigen adjuvant, or with PBS as a control. After the second immunization, blood was collected from the tail veins. Then, mice were challenged intranasally with 10^6^ PFU of RSV rA2-Line19F-FFL. *In vivo* imaging of lungs was performed daily for 5 days following RSV infection. Mice were sacrificed on day 5 post-challenge, when lungs and blood were collected. (B) Serum samples were collected from individual mice after second immunization and antibodies were analyzed using ELISA plates coated with purified RSV rA2-Line-19F-FFL. The serum samples were serially diluted and the detection of antibodies were measured by absorbance at 490 nm. The mean + SD absorbance for each dilution is indicated. (C) End-point titers of the serum samples were determined as the reciprocal of the highest dilution providing an optical density (OD) twice that of the negative control. (D and F) Live whole body imaging was performed daily to detect firefly luciferase activity in the lungs (D) and nasal cavity (F) on day 5 post challenge. Graphs represent the quantification of total flux (photons). 50% and 30% of the mean flux value of the PBS control group is indicated by the two dotted lines. The lowest grey line labeled ‘UC’ represents the average flux values of the uninfected control mice (3.74 x 10^4^ for lungs and 1.48 x 10^4^ for nasal cavity). (E) Lung viral loads at 5 days post-viral challenge as measured by plaque assay. Statistical significance was tested by one-way ANOVA and Bonferroni multiple-comparison tests. ****, *p* <0.0001; ***, *p* <0.001; **, *p* <0.01 *, *p* <0.05. (G-I) Correlation between ELISA endpoint titer (Log2) and bioluminescence flux signal in lungs (G) or nasal cavity (I) on day 5 post-challenge, or viral load measured by plaque assay in lungs on day 5 post-challenge (H). The symbol colors correspond to the groups shown in (D-F). Inverse Spearman correlations were observed between endpoint titers measured by ELISA vs. Lung Fluxes (r = -0.2935; *p = 0*.*1223*) (G) and vs. Lung viral loads (r = -0.4835; *p = 0*.*0053*) (H) and vs. Nasal Cavity Fluxes (r = -0.0532; *p = 0*.*0*.*7880*) (I).

Murine immunization with KLH-conjugated G peptides (Fig 5A) elicited antibodies that bound RSV A2 virions at various levels, but did not reach the binding titers elicited by the REG 67–298 ectodomain (Fig 5B and 5C)[21]. Interestingly, following challenge with RSV rA2-Line19F-FFL, the majority of immunized animals showed reduced viral loads in the lungs on day 5 compared with sham-vaccinated animals as measured by either Fluxes (Fig 5D) or PFU (Fig 5E). The reduction in lung viral loads measured using live imaging ranged between 40–78% of the sham (PBS vaccinated) control group, and reached statistical significance for most groups of immunized animals, apart from animals immunized with CCD peptide G 169–207 or the G 236–263 peptide (Fig 5D), suggesting less consistent control of virus replication after challenge in these two peptides vaccinated groups. However, in the PFU assay, all vaccinated groups had statistically lower viral loads compared with the control group (Fig 5E). The viral loads in the nasal cavity were similar between the control and vaccinated groups, but two animals in the group vaccinated with G 169–207 had elevated fluxes (Fig 5F). These were the same animals that also had elevated lung fluxes (Fig 5D), suggesting less consistent control of virus replication after challenge in this specific group. Correlation of the binding antibody ELISA end-point titers to RSV A2 virions with the viral load measurements in the lungs and nasal cavities (Fig 5G–5I) demonstrated significant inverse correlation only between ELISA endpoint titers and the lung viral loads measured in the plaque assay (Fig 5H, r = -0.4835, *p = 0*.*0053*).

**Fig 6:**
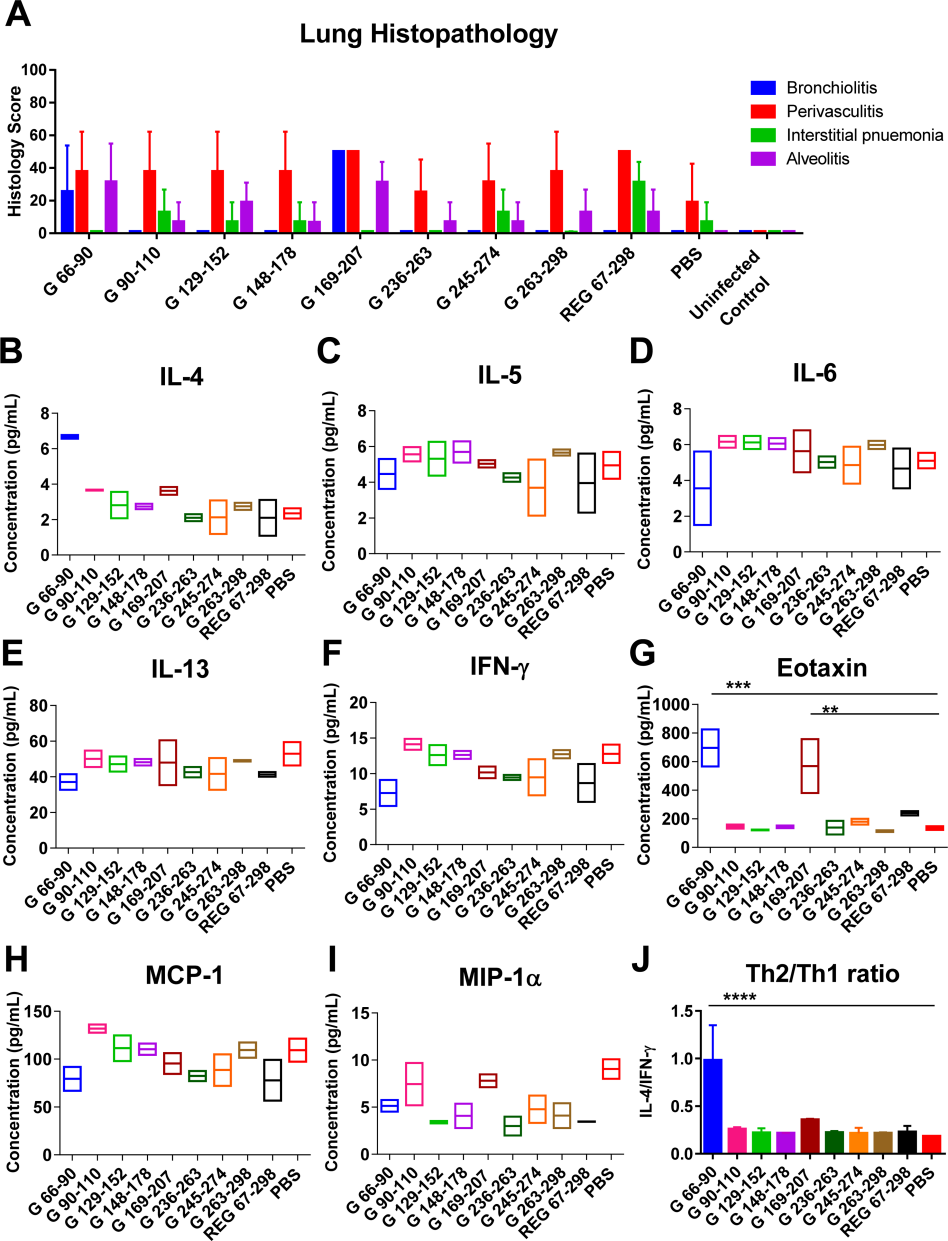
Histopathology and cytokine/chemokine levels in the lungs of animals vaccinated with the RSV G peptides at day 5 following RSV challenge. (A) Lung tissues were collected at 5 days post-challenge and were stained with hematoxylin and eosin. Individual lungs were scored for pulmonary inflammation: bronchiolitis (mucous metaplasia of bronchioles), perivasculitis (inflammatory cell infiltration around the small blood vessels), interstitial pneumonia (inflammatory cell infiltration and thickening of alveolar walls), and alveolitis (cells within the alveolar spaces). Slides were scored blindly using a 0–4 severity scale. The scores were subsequently converted to a 0–100% histopathology scales. (B-I) Levels of cytokines and chemokines in lung homogenates from day 5 post challenge were measured in a Bio-Plex Pro mouse cytokine assay. Values are cytokine/chemokine concentrations, expressed in picograms per milliliter. The mean for each group is shown. (J) Ratio of the observed concentrations of IL-4 vs. IFN*γ* shown in panels B and F. Statistical significance was tested by one-way ANOVA and Bonferroni multiple-comparison tests. ****, *p <0*.0001; ***, *p <0*.001; **, *p <0*.01 *, *p <0*.*05*.

In the same study, lung sections were evaluated for histopathology (Fig 6A) at day 5 post-viral challenge. The only notable finding was an increased bronchiolitis score in animals following RSV challenge that were vaccinated with G peptide 169–207, and to a lesser degree in some of the animals vaccinated with G peptide 66–90. Cytokine/chemokine profiling (Fig 6B–6I) revealed significantly higher levels of eotaxin in the lung extracts from the same two (G 66–90 and G 169–207) groups (Fig 6G). In addition, the Th2/Th1 cytokine ratio was 1 for the G 66–90 peptide vaccinated group, but well below 0.5 for all other vaccinated groups following RSV challenge (Fig 6J).

### Blocking of interaction between RSV G and CX3CR1 receptor by serum antibodies generated in rabbits against different G antigenic domains

**Fig 7:**
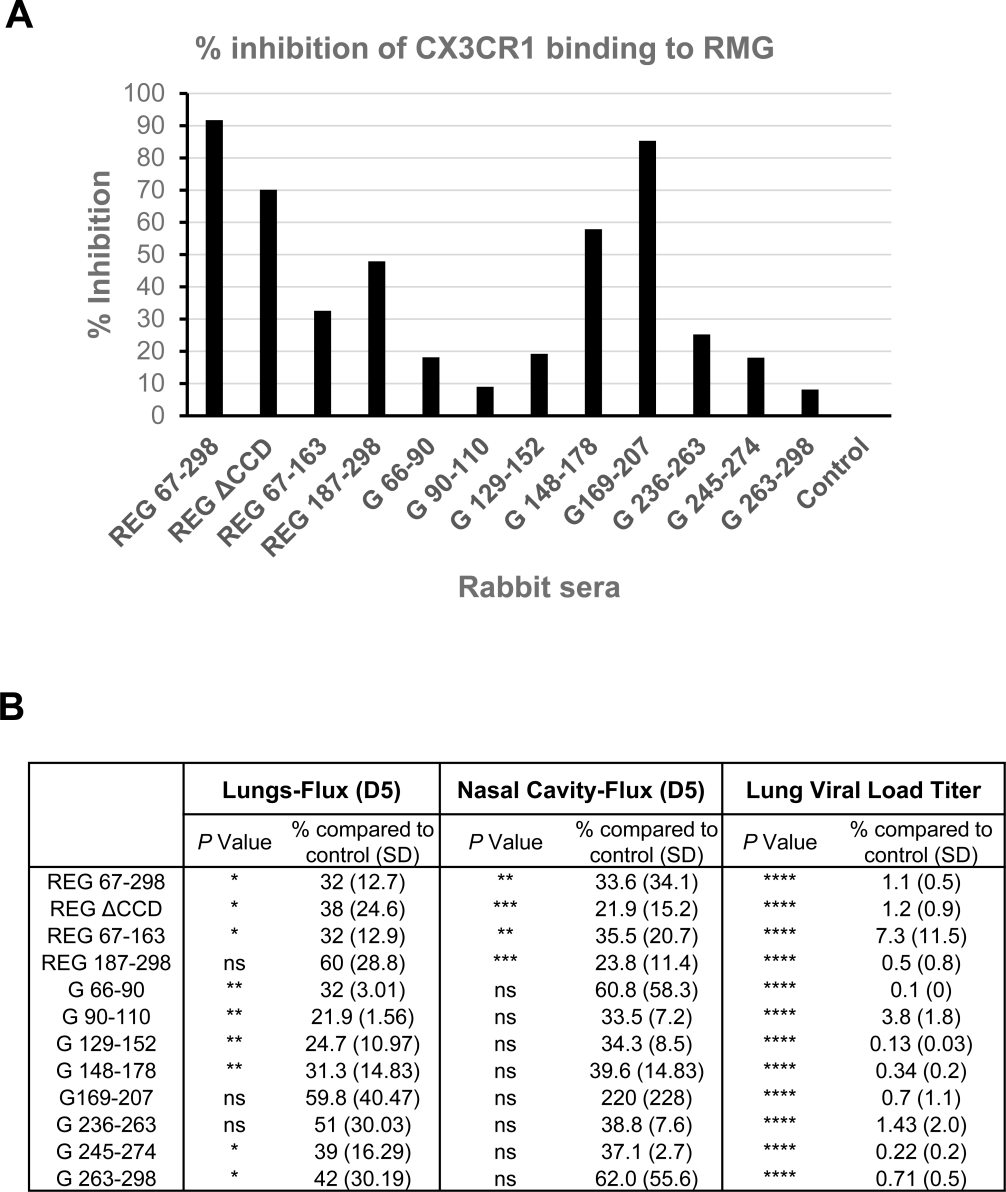
Serum antibodies generated against different G immunogens can block interaction between glycosylated RSV-G (RMG) and recombinant CX3CR1 receptor. (A) SPR assay was performed with the recombinant RSV-G protein from 293T cells (RMG) captured on a HTG sensor chip followed by addition of post-2^nd^ immunization rabbit sera (10-fold dilution). The binding of recombinant CX3CR1 to the antibody bound RMG was then measured by SPR. Pre-vaccination animal sera were used as a negative control. Total CX3CR1 binding to RMG in absence of any antibody binding was defined as 100%. Percentage inhibition for the pre- and post-vaccination rabbit sera was determined. (B) The mean flux values in lungs, nasal cavity, and the mean lung viral loads on day 5 post challenge of each group were compared to the PBS group set as 100%. Statistical significance when compared to the PBS group was tested by one-way ANOVA and Bonferroni multiple-comparison tests, ****, *p <0*.0001; ***, *p <0*.001; **, *p <0*.01 *, *p <0*.*05*; ns, not significant.

Since the *in vitro* neutralization assay does not reflect infection of human broncho-epithelial cells, which involves interaction between the RSV G protein and surface CX3CR1 [22–25], an SPR-based assay was performed to measure direct antibody-mediated blocking of recombinant CX3CR1 binding to glycosylated G protein produced in 293T cells (RMG) (Fig 7A).To that end, rabbit pre-vaccination and post 2^nd^ boost sera (at 10-fold dilution) were run on the chip captured with RMG prior to addition of rCXC3R1, and % inhibition was calculated for each serum sample. In this real-time binding assay, the strongest inhibition (90%) of CX3CR1-RMG interaction was observed with antibodies against complete REG ectodomain (67–298) followed by G 169–207 (85%), both of which contain the CX3C motif required for CX3CR1 binding[22]. Importantly, polyclonal antibodies elicited by the REG ΔCCD (aa 172–186 deleted), inhibited CX3CR1-RMG binding by 70%. In addition, short peptide (G 148–178) and the C-terminal antigenic domain (187–298) inhibited CX3CR1 binding by 48% and 55%, respectively (Fig 7A).

The degrees of conservation of the individual peptides used in our study among RSV A and RSV B strains are depicted in S1 Fig. and in S1 Table. The percentage homology was high among all RSV A strains but dropped significantly for RSV B strains. However, RSV A2 G peptides aa148-178 and aa169-207 showed ~70% conservation with RSV B1 and circulating B strains (S1 Table). Therefore, a G based protective vaccine may require a combination of G proteins from diverse RSV strains to protect against all RSV strains.

In summary, our study demonstrated that in addition to the central conserved domain (CCD) (aa 164–176), there are several protective antigenic sites within RSV-G, including the G- derived N- and C- sub-domains and linear peptides that were recognized by post-RSV infection human sera as previously reported [1]. Vaccination with these recombinant proteins and peptides provided at least partial protection in mice as measured by reduced lung viral loads on day 5 post challenge with RSV-A2 virus, with no significant lung pathology (Fig 7B).

## Discussion

The contribution of immune response against the RSV attachment protein G to either protection or potential enhanced disease have been well documented [8]. The G protein undergoes constant diversification in circulating RSV strains, and may contribute to the ability of the virus to reinfect throughout life [9–11]. Several monoclonal antibodies targeting the G protein were demonstrated to have protective activity against severe disease in animal models as well as anti-inflammatory effects [19, 26–29]. These MAbs bound to either linear or conformational epitopes overlapping and surrounding the CCD/cysteine noose CX3CR1 binding regions. Furthermore, levels of anti-G antibodies, in addition to antibodies against pre-F, were associated inversely with disease severity in RSV-infected infants and young children (<2yr) [14]. Therefore, dissecting the immune response to the G protein is important for better understanding of RSV viral immunity and the design of RSV G based vaccines [30].

In an earlier study, we used F- and G- phage display libraries (RSV-GFPDL) to dissect the antibody repertoire prior to and following primary RSV infection in infants. This analysis identified a large number of epitopes spanning the entire G ectodomain [1]. In this current study, we evaluated the ability of these linear and conformational antigenic sites within the RSV G attachment protein for their immunogenicity and ability to elicit protective immunity against RSV.

To address the contribution of G-CCD for protection against RSV, we compared full length recombinant RSV-G protein with and without the central conserved domain (CCD) (aa 172–186) in rabbits and mice. While virus-neutralizing antibody response to the CCD-deleted protein was significantly lower compared with the intact ectodomain, both proteins significantly protected mice from RSV challenge as measured by reduced viral loads in the lungs and nasal cavity. Lung viral loads were inversely correlated with ELISA titers of binding antibodies to RSV A2 virion particles. Furthermore, a low pathology scores and very low Th2/Th1 cytokine ratio was measured in the lungs of vaccinated animals on day 5 post RSV challenge, not significantly different from the sham–vaccinated control animals (Fig 3), suggesting avoidance of the severe lung pathology caused by FI-RSV vaccination [31].

It was previously shown that the CX3C motif downstream of CCD interacts with the CX3CR1 that serves as a receptor for RSV G protein on primary human airway epithelial cells [23]. Several MAbs targeting the CCD motif as well as MAbs 131-2G that binds to a motif upstream of the CCD were shown to block this interaction and neutralize RSV infection of airway broncho-epithelial cells, but not in traditional plaque neutralization assays [20, 23, 25, 26] and to protect mice from RSV challenge [19, 27]. In agreement, the antibodies generated against REG ΔCCD [with CCD residues 172–186 replaced by a (Gly_4_Ser)_2_ linker] in the current study, did not demonstrate significant virus neutralization *in vitro*, but provided significant *in vivo* protection.

We also investigated the immunogenicity of N- and C- subdomains of RSV-G and linear G antigenic sites that were identified by GFPDL screening of post- RSV exposure infant sera [1]. We found that the N- and C-subdomains (aa 67–163 and aa187-298) flanking the CCD motif generated antibodies that bound to fully glycosylated recombinant G protein produced in mammalian cells (RMG) using SPR, as well as to RSV virions in ELISA, but did not neutralize the virus in a PRNT assay. However, after RSV intranasal challenge of vaccinated mice, partial protection (i.e. reduction in lung and nasal virus loads on day 5) was observed (Fig 2). Furthermore, synthetic G-peptides containing mostly non-conformational antigenic sites, upstream and downstream of the CCD, also generated antibodies that bound the RMG [16] as well as intact RSV virions in ELISA (Figs 2 and 5). Since RMG protein and the G protein expressed on RSV virion particles (propagated in mammalian cells) are highly glycosylated, the antigenic sites outside the CCD are expected to be shielded from antibody recognition. Yet RSV-infected children clearly generated antibodies covering both conserved and less conserved sites in the G protein irrespective of predicted glycosylation levels, that could bind to the fully glycosylated G protein on virus particles as shown in our previous study[1] and was confirmed in the current study. Importantly, most animals in the challenge studies demonstrated mild lung pathology, and low Th2/Th1 cytokine ratios in lungs post-RSV challenge (Figs 3 and 6)

In a newly developed SPR-based real-time kinetics assay, we demonstrated inhibition of interaction between recombinant CXC3R1 protein and glycosylated RSV-G protein with sera from rabbits vaccinated with the G ectodomains, sub-domains, and linear peptides (Fig 7). The percentage of inhibition ranged between high (70–90%) for REG (67–298) REG ΔCCD, and G 169–207 peptide, medium (48–50%) for REG C-terminus domain (187–298) and G 148–178 peptide, or low (<30%) for all other peptides.

Surprisingly, G peptide (aa 169–207) spanning the cysteine noose and the conserved CCD motif, which was the immunodominant region recognized by post-RSV infection plasma from humans across multiple age groups [1], was not very protective in the current study (only 40% protection with large intragroup variability) (Fig 5D and 5F and Fig 7B). Interestingly, animals vaccinated with this peptide showed an increase in perivasculitis score and higher levels of eotaxin in lung extracts on day 5 post viral challenge (Fig 6A and 6G). It was earlier reported that atypical eosinophilia in RSV infected BALB/c mice was triggered by vaccination with G peptide (aa 184–198) in a CD4—dependent mechanism[32]. Another study using recombinant vaccinia virus (rVV) expressing G inserts identified residues 193–205 to be responsible for G-induced weight loss and lung eosinophilia in mice [33]. Both sequences are included in our G (169–207) peptide that was recognized by convalescent sera from infected children. Therefore, it is possible that this immunogen has the potential to induce both protective antibodies inhibiting interaction of RSV-G with the CX3CR1 and to promote proinflammatory environment in the form of high eotaxin secretion and eosinophilia. The role of CD4 T cells will need further investigation and may vary between mice and humans.

Taken together, these data suggest that in addition to the highly conserved CCD region, other antigenic sites in the G protein may contribute to protection against RSV in animal models and possibly humans[21]. The mechanisms of protection mediated by G antibodies needs to be further investigated. The PRNT assay in A549 cells is clearly not optimal for detection of all protective anti-RSV G antibodies. It is likely that antibodies elicited by some of the G domains and linear peptides would block RSV infection *in vivo* by directly blocking the G protein-CX3CR1 receptor interaction on lung broncho-epithelial cells. In addition, antibodies to other regions of RSV-G could mediate protection by other effector mechanisms including antibody dependent cellular cytotoxicity (ADCC) and antibody-dependent cellular phagocytosis (ADCP) that could contribute to removal of RSV infected cells *in vivo* [20]. We also cannot exclude contribution of cell mediated immunity by RSV-G specific CD8^+^ T cells. However G protein lacks MHC class I-restricted epitopes and has not been shown to elicit CTL responses in mice or humans [34, 35]. It was reassuring that the REG ΔCCD construct elicited a favorable ratio of Th1/Th2 cytokines, different from the formalin inactivated killed RSV vaccine that skewed the immune system towards Th2 responses and showed enhanced disease in naïve humans and animals after virus exposure.

In earlier studies, Powers *et al*. described the immunogenicity of bacterially produced fusion protein, BBG2Na that contained the central conserved domain of RSV-A2 G (aa 130–230) (G2Na) fused to the albumin-binding domain of streptococcal protein G (BB) formulated with aluminum adjuvant [36, 37]. Three of our peptides overlap with the G sequence in BBG2Na and our data are in general agreement with these studies. However, our REG immunogen encompass the entire G ectodomain with no “foreign/fusion” sequence that may re-direct the immune responses following vaccination.

In summary, the current study evaluated the immunogenicity of multiple antigenic sites within the RSV G protein and shows for the first time the presence of *in vivo* protective epitopes outside the CCD conserved motif. This information can help to explain findings in RSV exposed individuals and could contribute to further development and evaluation of safe and effective G protein based vaccine against RSV.

## Materials and methods

### Cell, viruses, and plasmids

A549 cells (Cat. No. #CCL-185) were obtained from the American Type Culture Collection (ATCC, Manassas, VA, USA) and were maintained in F-12K medium supplemented with 10% fetal bovine serum, 1X penicillin streptomycin (P-S), and L-glutamine. Cells were maintained in an incubator at 37°C under 5% CO_2_. RSV rA2-Line19F-Firefly Luciferase (rRSV-A2-L19-FFL) expressing the firefly luciferase gene upstream of the NS1 gene was prepared by infecting sub confluent A549 cell monolayers in F-12K medium supplemented with 2% FBS and 1X Penicillin-Streptomycin (infection medium) [15]. To generate a challenge stock, at 5 days post infection (dpi), cells were freeze-thawed twice and virus was collected. Harvested viruses were cleared of cell debris by centrifugation at 3,795g for 15 min. Virus stocks used in challenge studies were pelleted by centrifugation at 10,509g overnight. Pelleted virus was resuspended in TEN buffer and purified by sucrose-gradient ultracentrifugation. Virus titers were determined by plaque assay on A549 cells. The optimal challenge dose (10^6^ PFU intranasally) was determined in earlier study in which viral loads were measured by traditional plaque assay, by qRT-PCR and by live imaging (flux) and gave comparable results in terms of viral kinetics and peak values [15]. Codon-optimized RSV G coding DNA for *E*. *coli* was chemically synthesized. *Not*I and *Pac*I restriction sites were used for cloning the RSV A2 G ectodomain coding sequence (amino acids 67 to 298) into the T7-based pSK expression vector for bacterial expression. DNA coding REG 67–163 and REG 187–298 were amplified by PCR using primers containing *Not*I and *Pac*I restriction sites. DNA coding REG ΔCCD with residues 172–186 deleted and replaced with a (G4S)_2_ linker was prepared by a two-step overlapping PCR. The deleted sequence contains the cysteine noose in addition to the CX3CR1 binding motif present in all RSV G proteins. All amplified DNA was digested with *Not*I and *Pac*I and ligated into the T7-based pSK expression vector for bacterial expression.

### Production of recombinant *E*. *coli* expressed G (REG) proteins

Recombinant RSV G 67–298 (REG 67–298), REG 67–163, REG 187–298, and REG ΔCCD proteins were expressed in *E*. *coli* BL21(DE3) cells (Novagen) and were purified as described previously [16, 17]. Briefly, REG proteins expressed and localized in *E*. *coli* inclusion bodies (IB) were isolated by cell lysis, denatured and renatured in redox folding buffer followed by dialysis. The dialysate was purified through a HisTrap FF chromatography column (GE Healthcare). The protein concentrations were analyzed by bicinchoninic acid (BCA) assay (Pierce), and the purity of the recombinant G proteins from *E*. *coli* (REG) was determined by SDS-PAGE. Linear peptides were synthesized chemically using Fmoc chemistry, purified by HPLC, conjugated to KLH, and dialyzed.

### Production of recombinant glycosylated G protein using 293 Flp-In cells (RMG)

The 293-Flp-In cell line (Cat. No. #R75007; ThermoFisher Scientific) stably expressing the RSV A2 G protein with secretory signal peptide from IgG kappa chain was developed as described previously (15). Briefly, 293-Flp-in cells were co-transfected with the plasmids expressing Flp-in recombinase and the RSV A2 G ectodomain in DMEM media (Invitrogen). Twenty-four hours after transfection, culture medium was replaced with fresh DMEM containing 150 μg/mL of hygromycin for selection of stably transfected cells. For protein expression, cells were maintained in 293-Expression media (Invitrogen), and culture supernatant was collected every 3–4 days. Supernatant was cleared by centrifugation and filtered through a 0.45 μm filter before purification through a His-Trap FF column (GE healthcare).

### Gel filtration chromatography

Proteins at a concentration of 2 mg/ml were analyzed on a Superdex 200 Increase 10/300 GL column (GE Healthcare) pre-equilibrated with phosphate-buffered saline (PBS), and protein elution was monitored at 280 nm. Protein molecular weight (MW) marker standards (GE Healthcare) were used for column calibration and for the generation of standard curves to identify the molecular weights of each purified protein.

### Plaque reduction neutralization test (PRNT)

For the plaque reduction neutralization test (PRNT), heat-inactivated serum was diluted 4-fold and incubated with RSV-A2 virus (diluted to yield 20–50 plaques/well) containing 10% guinea pig complement (Rockland Immunochemical; Philadelphia, PA, USA) and incubated for 1 h at 37°C. After incubation, 100 μl of the antibody-virus mixtures were inoculated in duplicate onto A549 monolayers in 48-well plates and incubated for 1 h at 37°C. Inoculum was removed prior to adding infection medium containing 0.8% methylcellulose. Plates were incubated for 5 to 7 days at which time the overlay medium was removed and cell monolayers fixed with 100% methanol; plaques were detected by immunostaining with rabbit RSV polyclonal anti-F sera (14), followed by addition of alkaline phosphatase goat anti-rabbit IgG (H+L) (Jackson) antibody. The reactions were developed by using Vector Black Alkaline Phosphatase (AP) substrate kit (Vector Labs, Burlingame, CA). Numbers of plaques were counted per well and the neutralization titers were calculated by adding a trend line to the neutralization curves and using the following formula to calculate 50% endpoints: antilog of [(50+y-intercept)/slope].

### Ethics statement

All animal experiments were approved by the U.S. FDA Institutional Animal Care and Use Committee (IACUC) under Protocol #2009–20 (mice) and #2008–10 (rabbit). The animal care and use protocol meets National Institutes of Health (NIH) guidelines.

### Rabbit immunizations

Female New Zealand white rabbits (KBL(NZW)BR strain from Charles River Labs) were immunized three times intramuscularly (i.m.) with 100 μg of each purified REG protein combined with Emulsigen adjuvant, or with KLH-conjugated RSV-G peptides combined with Emulsigen, every 28 days. Blood was collected 8 days after each immunization.

### Murine immunization, RSV challenge, and sample collection

Four- to 6-week-old female BALB/c mice (BALB/cAnNCr strain code #555) were obtained from the Charles River Labs. Mice [N = 6–8 per group] were immunized intramuscularly (i.m.) at day 0 and day 20 with 20 μg of purified REG protein or with 25 μg of KLH-conjugated peptides combined with Emulsigen adjuvant at total volume of 100 μl. Blood was collected from the tail vein on days 0, 14, and 30. On day 34, mice were anesthetized with isofluorane through inhalation according to mouse body weight and infected intranasally (i.n.) with 10^6^ PFU of rRSV-A2-L19-FFL as previously described [15]. Mice were sacrificed by CO_2_ asphyxiation 5 days post-RSV challenge (previously determined to be the day with peak viral load), and blood and lungs were collected. For determination of the viral load and cytokine analysis, the left lobe of the lung was collected.

### *In vivo* imaging of RSV-infected mice

Whole body live imaging of infected mice was performed using IVIS imaging system as previously described [15]. In brief, mice were anesthetized in an oxygen-rich induction chamber with 2% isoflurane and administered 20 μl of RediJect D-Luciferin bioluminescent substrate (Perkin Elmer) intranasally. After a 5-min interval, mice were placed in the IVIS 200 Imaging systems (Xenocorp) equipped with the Living Image software (version 4.3.1.). Bioluminescence signals were recorded for 2 min for whole body and for 1 min for lungs and nasal cavities, respectively. Images were analyzed with the LivingImage 4.5 software (PerkinElmer) according to manufacturer’s instructions.

### Determination of viral loads in lungs

Lungs (unperfused) were weighed and homogenized in F-12K-2% FBS-1X P-S (5 ml medium/g of lung) using an Omni (Kennesaw, GA) tissue homogenizer. The supernatant was cleared by centrifugation at 3,795 xg for 10 min and was used immediately for viral titration by plaque assay in A549 cells as described above.

### Measurement of cytokine levels in lungs

All lungs were weighed and homogenized in 5 ml of medium/g of lung, as described above, to normalize the amount of lung tissue used per sample. Homogenized lungs were further diluted in infection culture medium containing a 2X concentration of Complete EDTA Free protease inhibitor cocktail (Roche, Basel, Switzerland) and were used in a Bio-Plex Pro mouse cytokine 23-plex assay according to the manufacturer’s recommendations. Plates were read using a Bio-Plex 200 system (Bio-Rad, Hercules, CA).

### RSV ELISA

Immulon 2 HB 96-well microtiter plates were coated with 100 μl of purified RSV rA2-Line19F-FFL or RSV B1 virus in PBS (10^4^ pfu/well) per well at 4°C overnight. After blocking with PBST containing 2% BSA, serial dilutions of mouse serum in blocking solution were added to each well, incubated for 1h at RT, followed by addition of 2,000-fold dilution of HRP-conjugated goat anti-mouse IgG-Fc specific antibody, and developed by 100 μl of OPD substrate solution. Absorbance was measured at 490 nm.

### Surface plasmon resonance (SPR)

Steady-state equilibrium binding of post-vaccination animal sera was monitored at 25°C using a ProteOn surface plasmon resonance (SPR) biosensor (Bio-Rad). The recombinant G protein from 293T cells (RMG) was coupled to a GLC sensor chip via amine coupling with 500 resonance units (RU) in the test flow channels. Samples of 100 μl of freshly prepared sera at a 10-fold dilution or MAbs (starting at 1 μg/ml) were injected at a flow rate of 50 μl/min (contact duration, 120 seconds) for association. Disassociation was performed over a 600 seconds interval. Responses from the protein surface were corrected for the response from a mock surface and for responses from a buffer-only injection. Pre-vaccination animal sera were used as a negative control. Total antibody binding and data analysis results were calculated with Bio-Rad ProteOn Manager software (version 3.0.1).

### SPR based CX3CR1 binding and inhibition assay

The recombinant G protein from 293T cells (RMG) was captured on a HTG sensor chip via Histidine tag with 500 resonance units (RU) in the test flow channels. Samples of 500 μl of freshly prepared post-2^nd^ immunization rabbit sera at a 10-fold dilution were injected at a flow rate of 50 μl/min (contact duration, 300 seconds) for association. Following antibody binding, recombinant CX3CR1 (Abnova; 5 μg/mL) was injected at a flow rate of 50 μl/min (contact duration, 120 seconds) for association. Responses from the protein surface were corrected for the response from a mock surface and for responses from a buffer-only injection. Pre-vaccination animal sera were used as a negative control. Total CX3CR1 binding and data analysis and % inhibition by immune sera were calculated with Bio-Rad ProteOn Manager software (version 3.0.1).

### Statistical analysis

The statistical significances of group differences were determined using one-way analysis of variance (ANOVA) and a Bonferroni multiple-comparison test. Correlations were calculated with a Spearman two-tailed test. *P* values less than 0.05 were considered significant with a 95% confidence interval.

## Supporting information

S1 Fig Sequence alignment of RSV G ectodomain from diverse RSV strains. Strains include A2, A-Tracey, A2001-2-20, A/Riyadh/2009, A1998-12-21, Long, RSV-12, A-Bernett-61, Memphis-37, ON67-1210A, B1, B-CH18537, and BA/3833/99. RSV G peptides that were used to immunize rabbits and mice are displayed in black and gray lines.
(TIF)

S1 Table Sequence identity (%) of each antigenic site to different RSV strains. Numbers were calculated using Sequence Identity Matrix function in BioEdit.
(TIF)
