# Supplementary figures and images for: Protective antigenic sites in respiratory syncytial virus G attachment protein outside the central conserved and cysteine noose domains
Source: PLoS Pathog. 2018 Aug 24;14(8):e1007262. doi: 10.1371/journal.ppat.1007262 (PMC6126872; doi:10.1371/journal.ppat.1007262)

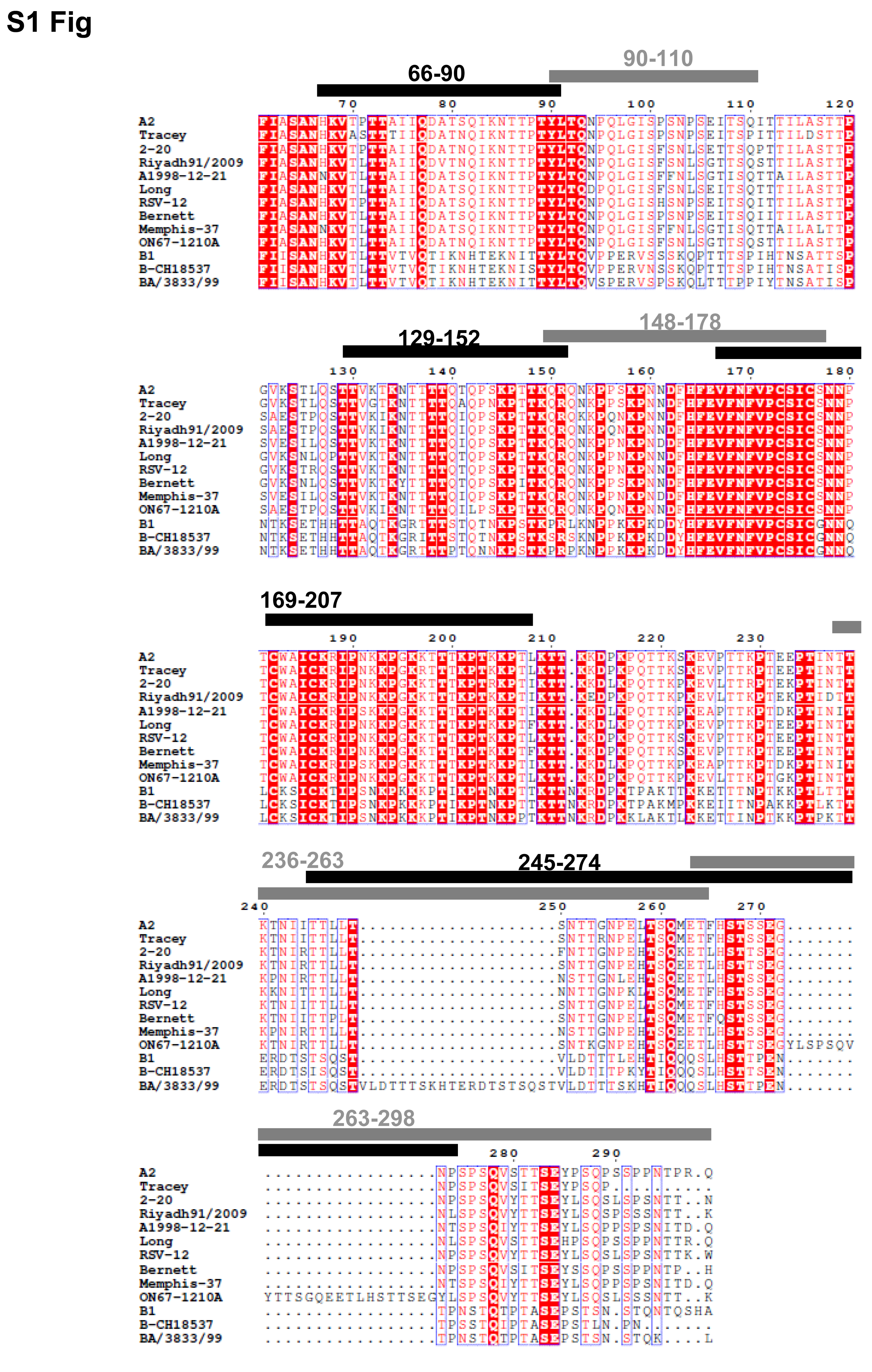

Supplement: S1 Fig — Strains include A2, A-Tracey, A2001-2-20, A/Riyadh/2009, A1998-12-21, Long, RSV-12, A-Bernett-61, Memphis-37, ON67-1210A, B1, B-CH18537, and BA/3833/99. RSV G peptides that were used to immunize rabbits and mice are displayed in black and gray lines. (TIF) [file ppat.1007262.s001.tif]

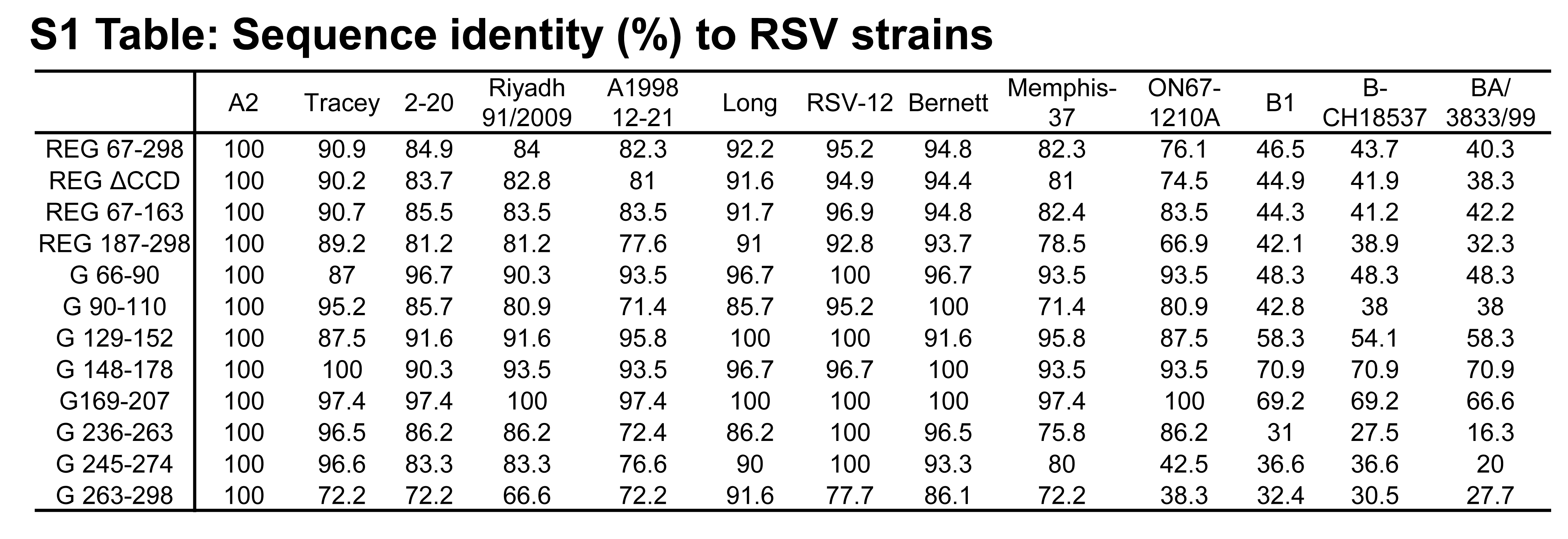

Supplement: S1 Table — Numbers were calculated using Sequence Identity Matrix function in BioEdit. (TIF) [file ppat.1007262.s002.tif]
